# “When You Thought That There Is No One and Nothing”: The Value of Psychodrama in Working With Abused Women

**DOI:** 10.3389/fpsyg.2018.01518

**Published:** 2018-08-23

**Authors:** Mihaela D. Bucuţă, Gabriela Dima, Ines Testoni

**Affiliations:** ^1^Department of Psychology, The Faculty of Social and Human Sciences, “Lucian Blaga” University of Sibiu, Sibiu, Romania; ^2^The Faculty of Sociology and Communication, Transilvania University of Brasov, Brasov, Romania; ^3^Department of Philosophy, Sociology, Pedagogy and Applied Psychology, University of Padova, Padova, Italy

**Keywords:** psychodrama, abused women, victim role, change process, interpretative phenomenological analysis

## Abstract

This paper discusses how psychodrama methods and techniques can empower abused women and stimulate changes in their victim role. Through an in-depth exploration, we sought to gain an insider’s perspective of the experiences of change and perceived outcomes for abused women, which could contribute to optimizing gender violence intervention. Theoretically, the study is grounded in the female co-responsibility and *trans*-generational transmission of women’s victim role from mother to daughter. A mixed methods experimental design employing an explanatory sequential approach to data collection was implemented. A total sample of 33 abused women (15 in the experimental group, and 18 in the control group) was involved in studying the impact of a psychodrama intervention combined with an ecological intervention. Spontaneity and wellbeing, considered in this study as dimensions of empowerment, were measured. Phenomenological interviews were conducted with 7 women 3 months after the psychodrama intervention ended, and with 6 women 5 years later. Data was analyzed using the Interpretative Phenomenological Analysis method. The matrix of themes that emerged reflects four overarching themes: the victim, the group experience, the process of change, and the corollary of change. Benefits perceived by the women include trust, hope, increased self-esteem, empowering, and courage to make decisions and changes. Findings describe three paths of change for women who participated in an empowering-oriented psychodrama intervention program: the Proactive – Resilient type, the Active – Resistant type, and the Repetitive – Non-Resilient type. Role-reconstruction and the interruption of trans-generational victim pattern were clear for the proactive type and possible for the active type, while the repetitive type showed minor changes but remained stuck in the victim pattern. As no claims to generalizability can be made, further research is needed to verify the proposed typology on larger samples. Psychodrama, as an action method, can empower abused women and has the potential to stimulate action in women’s lives and initiate adaptive coping strategies leading to resilience. The study ends with several suggestions for assisted resilience specialists.

## Introduction

Domestic violence is a complex phenomenon which cuts across all social classes, countries and periods of human history. It is one of the most widespread forms of violence among people, and it affects women in particular. If fundamental changes are to take place in women’s condition, they themselves must take an active role (World Economic Forum, 2012). The European Convention on Preventing and Combating Violence Against Women and Domestic Violence (Istanbul Convention) has provided a gender analysis framework that explicitly recognizes domestic violence as a serious violation of human rights and has thus become one of the most important instruments for tackling this social aberration and making Europe a safer place (Council of Europe, 2011). In addition, a number of European civil society networks have joined forces in the European Coalition to end violence against women and girls to raise awareness of the fact that certain women face a greater risk of violence because of their cultural background, where religious and traditional models are still core factors (European Women’s Lobby, 2017). Those factors have crossed our history like underground rivers that resurface just when they seem to have disappeared for good. Indeed, as Faludi (1991) points out, there has been a wide backlash against women lately, a backlash that has not been restricted to the United States, but also affects many other countries in the Western world. In Faludi’s view, the halting progress toward women’s social and political emancipation thorough extensive affirmative action programs has been a sequence of two steps forward and one step back. Resentment of female affirmative action has been matched by resistance to this democratic development. Both social (macro) and personal (micro) forms of backlash are cultural in origin and derive from a basic prejudice against women. Among the European Eastern countries in particular, the post-1989 transition from socialism to capitalism and democracy has produced a substantial regression in women’s condition. The erosion of social rights after the collapse of the socialist state has been accompanied by an anti-minority rights backlash, while many capitalist or conservative politicians have sought to reinforce class, gender, and race privilege, in line with the more traditional Western patriarchal model (Metcalfe and Afanassieva, 2005). Today more than ever, Eastern Europe women are seen as sexual objects. This objectification and the habit of regarding women as men’s property can be seen as root causes of such phenomena as trafficking, domestic violence, rape, sexual harassment and verbal abuse (Kruckenberg, 2010; Coleman and Sandfort, 2014). In Romania, despite the European Union’s policy efforts to solve those problems, this backlash has increased women’s long-standing submission to traditional values, to the opposite gender and, all too often, acts of violence against them.

Several Romanian studies have drawn attention to the country’s high tolerance of domestic violence in all its forms (Muntean and Munteanu, 2011). The most recent statistical report by the Romanian police shows that 16,122 cases of violence against women were recorded in 2017 (Reteaua Pentru Prevenirea Si Combaterea Violentei Impotriva Femeii, 2018). We can assume, however, that the real numbers are much higher. The findings of the European Union Agency for Fundamental Rights [FRA], 2014 survey on violence against women across the 28 member states indicate that only 17% of the Romanian respondents reported their most serious incident of violence to the police, and only 1% turned to social services, while for Europe as a whole, around 33% of victims contact the police and social services on average (European Union Agency for Fundamental Rights [FRA], 2014). Although there is sufficient evidence that Romania is still not able to comply with international provisions regarding the efficient protection of victims and implementing services for them (Adorjani, 2012), significant steps have been made in developing social policy, community responses and training specialists in the field (Dima and Beldianu, 2015).

### The Intergenerational Mandate and the Eastern Europe Backlash

Any social program that hopes to change the current situation must first change the cultural premises and associated stereotypes, since culture is often responsible for the way women and the issue of violence against them are viewed and addressed. We believe that domestic violence occurs as a result of the backlash that seeks to maintain and/or restore the traditional separation between natural and social tasks.

One of the world-wide effects of this backlash has been to reinforce sex and maternal roles, which weigh more heavily on Eastern Europe women than in the past (Occhipinti, 1996; Saxonberg and Sirovatka, 2008). The most important effect is that women’s expectations of getting married and becoming a mother are now stronger, which has a huge influence on their relational condition and existential choices. From this perspective, as was mentioned during the Beijing Conference, the mother-daughter relationship plays an essential role (Kaplan, 2001). The intergenerational mandate from mother to daughter and the related patterns of reproduction can inhibit young girls’ individuation processes because of this patriarchal influence. “Just take it as it comes” is the traditional way mothers have taught their daughters to resign themselves to traditional social and intimate oppression, abandoning aspirations to a mature agency and a better life (Meyers, 2001; McLeod, 2015). Indeed, as DiQuinzio (1999, 2013) shows, mothering requires consideration of women’s difference, since it jeopardizes feminism’s claims for women’s equal individualist subjectivity, and risks recuperating the inequality and oppression of women, especially the view that all women should be mothers, want to be mothers, and are most happy being mothers. In every culture, motherhood is often associated with female achievement; women are still under severe pressure to become mothers and to bear children, and thus accept that childbearing is a natural and necessary part of their life. As this leads to an idealization of mothering as an extension of emphasized femininity, women who are socially employed may feel guilty or be accused of selfishness when they pursue goals which disregard the primary duty of motherhood. The pressure on women to bear children does not derive merely from personal viewpoints, but draws mostly on cultural symbolism, which is transferred through the mother–daughter relationship, and thereby influences women’s individual attitudes.

### The Empower Project and the Present Study

Against this backdrop, the Empower Daphne Project (2011–2012; Testoni et al., 2013b) addressed the specific role of motherhood in domestic violence. This action research project conducted in Romania, Italy, Austria, Portugal, Bulgaria, and Albania aimed to empower women who fall victims of violence, mobilizing their coping strategies for greater resilience. By using psychodrama and active methods, the project made women aware of how the dynamics of their role and position in society had influenced their own lives, enabling them to change their situation. The victims were encouraged to rewrite their life experiences through psychodrama and story-telling.

Psychodrama has a significant advantage as a means of changing behavior, since it is based on theories of action, spontaneity and creativity (Dayton, 2013). It engages the person holistically: body, mind and emotions. On the psychodrama scene, the protagonist enacts his/her inner world, exploring parts of it with the support of the other group members, who play their assigned roles (auxiliary ego) (Moreno, 2009). Psychodrama offers a living laboratory in which former victims, in a safe clinical environment, have the chance to contemplate and experience their own lives, meet themselves, their perceptions of self and their relational experience. It enables women to process the roles taken on and change behavior through “exploratory, healing role play and role training” (Dayton, 2013, p. 6). Psychodrama aims to access the experience of spontaneity in order to produce new and creative solutions to old problems (Kipper, 1998). These techniques emphasize the “changing role,” which is a key element in promoting resilience.

### Research Aim and Questions

The main aim of this paper is to illustrate how psychodrama methods and techniques empowered and stimulated changes regarding the victim role of abused women who participated in a psychodrama intervention program. The focus is on an in-depth exploration in order to offer an insider’s perspective of the experiences of change of abused women which could contribute to optimizing gender violence intervention.

The research question is:

How do the data from interviews with abused women about their group experiences and perceived outcomes help explain the results of a psychdrama intervention program focusing on empowerment?

## Materials and Methods

A mixed methods experimental design employing an explanatory sequential approach to data collection was implemented (Creswell and Plano Clark, 2018, p. 199).

**FIGURE 1:**
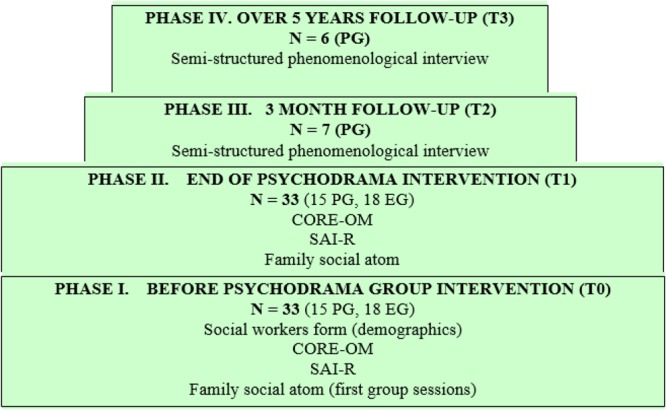
Research phases and data collection.

The quantitative component was part of the Empower Daphne Project (2011–2012; Testoni et al., 2013b) (**Figure 1, phases I** and **II**, November 2011 – June 2012) and consisted of an experimental design (*N* = 33; two experimental groups: *n* = 15, control group: *n* = 18) that investigated the impact of psychodrama group intervention on spontaneity and wellbeing.

The qualitative dimension of the study consisted of an in-depth exploration of the group experiences and perceived outcomes for abused women 3 months after the psychodrama intervention had ended (phase III, September – October 2012), and again 5 years later (phase IV, October – December 2017). The Interpretative Phenomenological Analysis Method (IPA) (Smith, 1996) was chosen for its two complementary commitments – ‘giving a voice’ and ‘making sense’ – which provide a platform for gaining an ‘insider’s perspective’ (Larkin et al., 2006). Shaw (2001, p. 48) argued that IPA is particularly suitable for investigations that concern the *“*uniqueness of a person’s experiences, the way experiences are made meaningful and how these meanings manifest themselves within the context of a person, both as an individual and in their many cultural roles.”

### Participants

A convenience sample was used.

Access was granted through a voluntary organization, Home of Hope, which was a partner of the Romanian Association of Classical Psychodrama (ARPsiC) in the Empower Daphne Project. Home of Hope supports abused women through a project entitled “Preventing and Combating Domestic Violence” which uses the ecological intervention framework (Bucuţă et al., 2012).

The 2011–2012 Empower Daphne Project was carried out in accordance with the guidelines of the University of Padova Scientific and Ethics Committee.

The sample included a selection of women who were Home of Hope clients during July – November 2011. They were informed regarding the project and the voluntary nature of their participation and were given the choice of participating in either the experimental group or the control group. Informed consent was given in writing. The specialists from Home of Hope acted as contact persons for any questions regarding the study. Upon their voluntary decision, *n* = 20 women were included in the control group and *n* = 16 in the experimental group. One woman withdrew from the experimental group during the intervention. During data analysis, two participants from the control group were excluded because of incomplete data.

The total sample thus consisted of *N* = 33 abused women, *n* = 15 in the experimental group (Psychodrama Group – PG; PG1: *n* = 8; PG2: *n* = 7) and *n* = 18 in the control group (Ecological Group – EG). The age range was 19 – 62 years for the total sample (*N* = 33; *M* = 33.18; *SD* = 9.15), 19 – 45 years (*M* = 31.20; *SD* = 9.42) for the experimental group, and 21 – 62 years (*M* = 34.83; *SD* = 8.84) for the control group. All women had experiences of abuse. Except for two students, all participants had one to five children.

The experimental group was divided into two psychodrama groups: PG1 (*n* = 8), consisting of women living at home, and PG2 (*n* = 7) consisting of women living in a shelter for victims of abuse. The women in PG1 were followed up longitudinally. The sample profile is described in **Table 1**. Pseudonyms are used to maintain anonymity.

**Table 1:** Sample profile of the psychodrama group followed up longitudinally (PG1).

Participant No.	Age range	Marital status	Children	Education	Follow-up at 3 months	Follow-up over 5 years
1	20–25	Single	0	University	No	No
2	36–40	Divorced	2	High school	Yes	Yes
3	20–25	Single	0	University	Yes	Yes
4	40–45	Divorced	1	High school	Yes	Yes
5	40–45	Married	3	High school	Yes	Yes
6	36–40	Divorced	1	High school	Yes	No
7	36–40	Divorced	1	University	Yes	Yes
8	45–50	Divorced	1	University	Yes	Yes

### Procedures

The research phases and data collection are described in **Figure 1**. The quantitative component used social workers’ case information forms (demographic data), the Revised Spontaneity Assessment Inventory (SAI-R) and the Clinical Outcomes in Routine Evaluation Outcome Measure (CORE-OM). Qualitative data was collected using a semi-structured phenomenological interview.

The **Revised Spontaneity Assessment Inventory** (**SAI-R)** (Kipper and Shemer, 2006) is a questionnaire initially devised by Moreno to assess spontaneity, later completed and revised by Kipper and his colleagues. The SAI-R is designed to measure the intensity of the presence of spontaneity by posing one question: “*How strongly do you have these feelings and thoughts during a typical day*?” The question is followed by a list of 18 adjectives and phrases describing feelings and thoughts, which are rated on a five-point Likert scale from “1 = very weak” to “5 = very strong,” where higher scores indicate higher spontaneity.

The **Clinical Outcomes in Routine Evaluation Outcome Measure (CORE-OM)** (Evans et al., 2002) is routinely used as an initial outcome measure of wellbeing and treatment outcomes for individual patients. The CORE-OM contains 34 simply worded items answered on a five-point scale ranging from “not at all” to “most or all the time,” covering four areas: wellbeing, commonly experienced problems or symptoms, life or social functioning, and risk to self and others. A total score is also calculated. Overall, the measure is problem-scored, higher scores being indicative of more problems. The scale has good sensitivity to change.

Both SAI-R and CORE-OM were used to evaluate the clinical efficiency of the interventions and were cross-culturally validated in the Empower Daphne Project (Testoni et al., 2013a).

### Semi-Structured Interview

An interview schedule was formulated and applied in a flexible manner, using a phenomenological approach (Smith et al., 2009). Interviews ranged from 60 to 100 min in length. Participants were asked to give their written consent for the interview to be recorded.

The dimensions of the interview are:

1. Psychodrama group experience
2. Perceived impact of psychodrama techniques and activities
3. Perception of change
4. Perception of self
5. Messages for other victims of gender violence

In addition to these common dimensions, the 3-month follow-up (T2) included the individual and trans-generational history of abuse, while the 5-year follow-up (T3) explored significant experiences/events during this period.

### The Ecological Intervention

The World Health Organization [WHO] (2002) ecological approach to abuse conceptualizes interpersonal violence as a multifaceted phenomenon grounded in an interplay among many factors at four levels – the individual (e.g., personal history, biological factors), the relationship (e.g., intimate partners, family, friends, peers), the community (e.g., schools, neighborhoods, workplaces), and the societal (e.g., economic and social policies, social-cultural norms). This framework is useful to identify, and cluster intervention strategies based on the ecological level in which they act. The ecological intervention was carried out by social workers from Home of Hope and consisted of counseling oriented toward identifying needs and resources and building a support network around the person. Social actors such as health professionals, police officials, lawyers or child protection officers were involved.

### The Psychodrama Intervention

Psychodrama was used as part of a psycho-social intervention program aiming to empower abused women. Before the group started, the psychodramatist had one individual session with each member to prepare the women for the group and build therapeutic alliances. The psychodrama intervention program consisted of a total of 25 2-h sessions, provided on a weekly basis. When the project ended, Home of Hope offered follow-up after-care for those who still needed support. The group psychodramatist had 10 years of experience in using psychodrama and was supervised by the national Empower Daphne project coordinator/psychodrama trainer and supervisor to ensure treatment integrity and fidelity.

The sessions are summarized in **Table 2**, where they are grouped according to an adaptation of Tuckman’s group development stages (cited in Yalom and Leszcz, 2008): forming (sessions 1 – 6), norming (sessions 7 – 10), performing (sessions 11 – 21) and adjourning (sessions 22 – 25). The table presents the main objectives of the intervention and provides some examples of activities and the techniques used. Each session consisted of a warm-up, psychodramatic group or protagonist work, processing and sharing emotions and ending rituals.

**Table 2:** Summary of psychodrama sessions.

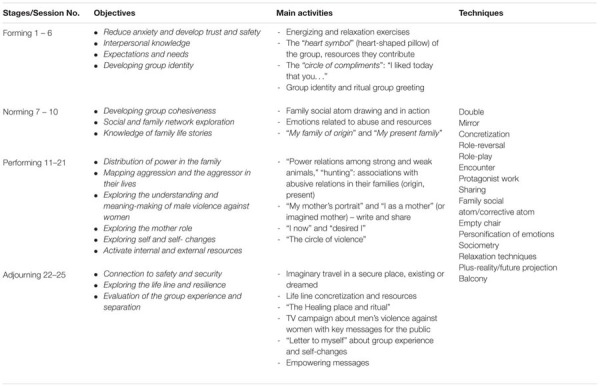

### Data Analysis

**Quantitative data** were analyzed using SPSS 21. Descriptives and reliabilities (standardized Cronbach’s alphas) were calculated. The non-parametric Wilcoxon signed rank test was used to compare mean ranks between pre- and post-test within the experimental group. Analysis of covariance (ANCOVA) was used to compare the experimental and control groups, while statistically controlling for the effects of the differences at pre-test.

**Qualitative analysis** was based on a total of 13 verbatim transcripts (7 from T2, 6 from T3). Each transcript was analyzed until an IPA matrix of themes emerged; common themes and discrepancies were searched for until a group matrix for T2 and one for T3 were generated. These two matrices were analyzed for consensual themes and one IPA matrix emerged, capturing the experiences of women longitudinally (Smith et al., 2009). This is the basis of the narrative report.

IPA results “reflect the researcher as much as the researched” (Brocki and Wearden, 2006, p. 99). Credibility was established by consulting two IPA analysts who discussed the intermediate matrices and agreed on the final IPA matrix of themes. A member check was carried out with one woman (Elliott et al., 1999).

### Quantitative Results

The reliabilities of the questionnaires (standardized Cronbach’s alphas) were excellent for the total sample (*N* = 33), for both SAI-R (T0: α = 0.94; T1: α = 0.93) and CORE-OM (T0: α = 0.93; T1: α = 0.93). Reliabilities calculated for independent samples, pre-test and post-test are also good and very good as detailed below:

1. Experimental group (*N* = 15): SAI-R (T0: α = 0.89; T1: α = 0.86) and CORE-OM (T0: α = 0.90; T1: α = 0.95)
2. Control group (*N* = 18): SAI-R (T0: α = 0.94; T1: α = 0.95) and CORE-OM (T0: α = 0.93; T1: α = 0.89)

Descriptive results presented in **Table 3** indicated that the difference between T0 and T1 with SAI-R points to an increase in spontaneity for both the experimental and the control group. The mean differences for CORE-OM between T0 and T1 show a tendency to decrease – denoting an increase in wellbeing – for both groups, indicating that both psychodrama and ecological interventions can support abused women in their recovery process.

**Table 3:** Descriptive data of spontaneity and wellbeing scores at T0 and T1.

			Pre-test (T0)		Post-test (T1)	
			*M*	*SD*	*M*	*SD*
SAI-R	Experimental		55.89	11.83	59.45	10.38
	Control		38.39	13.19	49.33	11.21
CORE-OM	CORE-OM Total	Experimental	51.68	13.03	45.67	13.15
		Control	61.22	13.10	52.71	11.49
	CORE-OM Wellbeing	Experimental	7.47	1.96	7.67	1.50
		Control	8.33	2.11	8.72	2.47
	CORE-OM Problems	Experimental	19.47	8.73	14.05	9.20
		Control	26.78	8.84	19.39	7.55
	CORE-OM Functions	Experimental	23.08	4.25	22.67	3.35
		Control	23.67	5.79	22.86	5.58
	CORE-OM Risks	Experimental	1.67	2.53	1.27	2.66
		Control	2.44	2.71	1.72	1.60
	CORE-OM Non Risk	Experimental	50.03	11.48	44.35	10.99
		Control	58.78	11.36	51.01	10.61

Furthermore, we analyzed the experimental group using the non-parametric Wilcoxon signed rank test. Results illustrated in **Table 4** show no statistically significant improvement in spontaneity (SAI-R) between pre- and post-test. For CORE-OM, the results for the Problems scale are statistically significant, indicating a decrease in experienced problems or symptoms; the mean score and standard deviation at pre-test were T0: *M* = 19.47, *SD* = 8.73, while at post-test T1: *M* = 14.05, *SD* = 9.20. Results for the Non-Risks items are marginally significant (*p* = 0.06), showing a tendency to decrease self-risk behaviors: the mean score and standard deviation at pre-test were T0: *M* = 50.03, *SD* = 11.48, while at post-test T1: *M* = 44.35, *SD* = 10.99. The total CORE-OM score is also marginally significant (*p* = 0.06), pointing to an improvement in wellbeing. Hence, the mean score and standard deviation at pre-test were T0: *M* = 51.68, *SD* = 13.03, while at post-test T1: *M* = 45.67, *SD* = 13.15 (**Table 3**).

**Table 4:** Wilcoxon signed rank test for experimental group (pre- and post-test results).

Scales	Experimental Group Wilcoxon Signed Rank Test	
	*z*	*p*^∗^
SAI-R Pre-test/Post-test	–0.99	0.16
CORE-OM Total Pre-test/Post-test	1.59	0.06
CORE-OM Wellbeing Pre-test/Post-test	–0.47	0.32
CORE-OM Problems Pre-test/Post-test	1.82	0.03
CORE-OM Functions Pre-test/Post-test	0.29	0.39
CORE-OM Risks Pre-test/Post-test	0.94	0.17
CORE-OM Non Risk Items Pre-test/Post-test	1.57	0.06

*^∗^*p* (one-tailed) was calculated for the uni-directional hypothesis.*

Analysis of covariance (ANCOVA) was used to compare the experimental and control groups, while statistically controlling for the effects of the differences at pre-test. Results presented in **Table 5** do not identify statistically significant differences between the experimental and control groups, as the control group also showed improvements between pre and post-test.

**Table 5:** Covariance analysis (ANCOVA) for experimental and control group.

Scales	ANCOVA	
	*F*	*p* (1-tailed)
SAI-R Pre-test/Post-test	0.64	0.43
CORE-OM Total Pre-test/Post-test	0.32	0.58
CORE-OM Wellbeing Pre-test/Post-test	1.05	0.31
CORE-OM Problems Pre-test/Post-test	0.65	0.43
CORE-OM Functions Pre-test/Post-test	0.02	0.89
CORE-OM Risks Pre-test/Post-test	0.00	0.96
CORE-OM Non Risk Items Pre-test/Post-test	0.51	0.48

*^∗^*p* (one-tailed) was calculated for the uni-directional hypothesis. *N* = 33: *n* = 15 experimental group, *n* = 18 control group.*

To conclude, the results show that it seems possible that psychodrama contributed to a positive trend of improving wellbeing, especially in terms of problems and risks. As regards spontaneity, the experimental group showed no statistically significant improvement compared to the control group.

However, while questionnaires were able to identify only minor differences between the improvements shown by women participating in the psychodrama program and those who only received ecological intervention, the observations and comments of the two psychodramatists indicated more complex changes. Further analysis was thus carried out based on phenomenological interviews.

## Qualitative Findings

This study does not claim to be representative; the main objective is to explore the processes in depth and create meaning. It centers on “giving voice” and “making sense” of women’s experiences, perceptions and views (Reid et al., 2005), thus gaining an insider’s perspective to help explain the results of the psychodrama intervention program focused on empowerment. Consequently, we aim to offer information which could contribute to optimizing gender violence intervention.

The matrix of themes that grounded the results is presented below (**Table 6**).

**Table 6:** IPA matrix of themes.

Theme	Subtheme
The Role of Victim	(1.1) The role of victim (1.2) The generational model

The Group Experience	(2.1) The difficulty of receiving help (2.1.1) Shame (2.2.2) Lack of trust (2.2) The relationship with the group therapist (2.3) The perceived impact of action methods and techniques

The Process of Change	(3.1) The basis of change - The perceived effects of psychodrama intervention (3.2) Relations (3.3.1) The relationship with one’s daughter or son (3.3.2) The relationship with one’s mother (3.3.3) The relationship with one’s life partner

Corollary of Change	(4.1) Decisions made over 5 years (4.2) Self-perception (4.3) The significance assigned to the psychodrama intervention

### Theme 1: The Victim: “Shut Up, Let It be and Suffer”

The first theme comprises the life experience of the participants, women who were victims of violence and establishes the context for the subsequent themes. It provides an answer to the question of *where* exactly the participants began the long and difficult pathway toward change.

#### 1.1. The Role of Victim

All participants in the study have their own experience of abuse and violence. All forms of abuse are present in the women’s life histories: from verbal to physical, sometimes even life-threatening abuse. One of the participants reports: “…he threw things at me, anything he could grab […] he seized me by the throat […]. And he threw me to the floor and spat on me” (Maria, 2012).

Many of them lived in terror: “that fear…that he would do it, that he would come and kill me...” (Diana, 2012). Silence, putting up with humiliation, terror, being careful not to provoke the perpetrator were all part of their survival strategies: “… I was silent and that bothered him more and he had all kinds of outbursts and I started being afraid that my life and my children’s were threatened...” (Maria, 2012).

The difficulty of ending an abusive relationship is common to all participants. The reasons invoked by most of them are the lack of financial independence, no place to go, no job, family pressure (especially the mother’s) and sometimes poor health: “…if only I had a job, or my health was better, I would have done otherwise...” confesses Maria. They all “gave another chance” to the abusive husband: “…I always gave him another chance. I thought he would change” (Mona, 2012).

#### 1.2. The Generational Model

With one exception (Mona), all participants in the study found themselves to be the third generation of victims of violence. Although they faced extreme violence, the great majority of participants’ grandmothers and mothers stayed in an abusive relationship. In trying to find an explanation, all participants invoke the same reasons: social pressure (Romanian patriarchal society), faith (religion), and motherhood. For example, one of the participants says: “poor dear... (Grandma) lived in a society in which nobody listened to you if you told them what you had to suffer as a woman; they would listen to the man…” (Maria, 2017). One generation later: “…she (mother) would have thought of divorcing, but at that time, Ceauşescu’s time, there was the shame of divorcing... kids without a father!” recalls Erna, 2017.

Ina explains her grandmother’s and mother’s decision to stay in the abusive relationship by invoking the religion, customs and mentality of those times: “their elders taught (grandma and mother) that the moment you marry someone and take the oath before the holy shrine, before God that you are going to be together, you are not allowed to leave as you are going to upset God... and she swore she would stay with him (grandpa) until death did them part, and she stayed, plus she had the children” (Ina, 2012).

Maria’s account is illustrative of how the victim model was transmitted from generation to generation: “She (grandma) was a fighter, there was no divorce or separation at the time […]. And then mother handed it down to me, this model of misplaced obedience. Yes, obedience, shut up and let it be and swallow it, a model mother took up somehow and handed it down to me... I used to live with this model, I took it with me in my marriage and... my daughters saw it for a while...” (Maria, 2012).

### Theme 2: The Group Experience: “Just When I Thought No One and Nothing Could Help Me”

#### **2.1.** The Difficulty of Receiving Help

All women stated that they had difficulty in asking for and receiving help.

The good relationship built up with the specialists (one psychologist and one social worker, both trained in psychotherapy) during the ecological intervention carried considerable weight in their decision to participate in the group.

##### 2.1.1. Shame

Group participation was initially accompanied by fear, anxiety, shame, holding back. Maria’s account illustrates the struggle with shame all the participants experienced in their lives: “Yes, I was ashamed of the situation I ended up in, what I knew about life, what I wanted my marriage to be like and where I was at the time… I was ashamed of those around me, of myself, of the choices I had made, of my family, my daughters, my mother and my brothers.” (Maria, 2012). For most of them, joining the group initially meant “to complain, to display all your problems” (Mioara, 2012) and accepting the fact that they cannot manage for themselves: “…I think I was simply ashamed to admit that I was in trouble, I thought that meant I was incapable (Erna, 2012).

##### 2.1.2. Lack of trust

The abuse experience led to mistrust the idea that someone might help them: “… if the man closest to you, the man you loved and offered the best you had could act like that, why would some strangers treat you better?” (Maria, 2012).

The conviction that they cannot be helped, that only they can help “by themselves” was unanimously shared: “Before I came to the group, I absolutely refused to believe that anyone else could help me… I have to help myself...” (Erna, 2012).

#### **2.2.** The Relationship With the Group Therapist: “She Came Close to Us in Steps of Smoke”

Fear, mistrust, shame were overcome only with the help of the group psychotherapist. The first meeting was perceived with considerable anxiety. All participants described the psychotherapist’s attitude as unobtrusive, affectionate, delicate and mindful; that was the only thing that engendered trust from the very beginning. Erna reports: “She treated me, us, the girls, in a very delicate way, and that brought me pleasure and a feeling of trust, belonging and self-value” (Erna, 2012).

Maria described the psychotherapists’ attitude with a metaphor: “…step by step, slowly, Ema (the psychotherapist) came closer to us in steps of smoke” (Maria, 2012).

Understanding, encouraging, and appreciating women’s efforts to participate in the group was a major part of the decision not to abandon the intervention program.

For most of the women, the relation with the psychotherapist was the first model of another type of relationship which generated a new experience.

For some women, the therapeutic relationship was the first experience of being accepted, valued and loved, which brought the light of hope into their life and motivated them to change: “and if Ema treated us with respect, dignity and patience – after all, this means love for the other – and I thought that if she (the therapist) wants to help me, a person I’d never met before, if God loves me, if the girls (the other participants in the group) agree to interchange […] that means that self-love exists and I have to find it and build it for me (Maria, 2017).

#### **2.3.** The Perceived Impact of Action Methods and Techniques

The feelings and emotions engendered by the psychodrama techniques were powerful and diverse: both positive and negative, and experienced, more than a few times, as strange and odd.

**Ludic, psychomotor activation and mental imagery activities** were perceived by the great majority of the participants as an antidote for anxiety and a way to facilitate self-confession: “…the initial relaxation games […] Yes, we were relieved and somehow we were able to open up more easily” (Paula, 2012). The connection with the present, with the “here and now” was another perceived effect of these activities, claims Ina: “there were little games and we focused effectively upon what happened here, and then negative outer things were left out there that moment...” (Ina, 2017). Moreover, they were among the first activities that allowed participants to access positive emotions: “an island of positive emotions, in a sea of negative emotions” (Mona, 2012).

**The Mirror**, another technique perceived as being helpful and having an impact, stirred powerful positive emotions and new experiences. “…and I received so much... it helped me remember who I can be” (Mona, 2012).

The activities that are most frequently cited as having the largest impact, both in 2012 and 2017, are **role-play** and **role-reversal**. For most participants, role-play is a chance to work on resistances, thus making action and interaction possible: “…that role-play, the interplay with the others… whether you want it or not, you interact, you cannot avoid it…” (Erna, 2012). Other times, it enabled them to build new experiences, which generated contents and new positive emotions. “The great benefit here is the fact that it helped me understand and to keep the hope alive that not all men are like that, that somewhere there is a good man....” (Maria, 2017).

The most difficult and disturbing experiences were engendered by role-reversal. “Inversions, well, disturbed me and moved me a great deal, but they helped me for the future, for the years to come, to understand her (the mother) and become closer” (Maria, 2017).

All women experienced the role of protagonist, that of auxiliary ego and the witness. The experience is described in terms such as “odd,” difficult, and engendering strong negative emotions. The women’s defenses and resistances were severely tested. Maria reports: “Emotions were very strong, sometimes I felt like quitting, although the voice of reason said that it was ok and there was a struggle between reason and sensibility, I was telling myself I’m not going there anymore, I can’t take any more of that pain...yet I continued to go, I came” (Maria, 2012). Another participant says: “It seemed weird to me the moment where my group colleague was, say, my mother or my daughter or my friend or my husband, the impact was large. There were moments when I cried, those when my hair stood on end, moments of very, very strong feelings” (Paula, 2012).

After 5 years, most of the participants appreciate that the most impactful and beneficial experiences are still those engendered by role-reversals and role-playing. For many of them, those experiences led to an increase in emphatic capacity, to improving the ability to identify and express one’s own emotions and thoughts, to a change of perspective: “…they were some of the toughest moments (the inversions with mother), it’s hard to be in the other’s shoes and...Yet this is how I started to understand and later to accept!”(Mona, 2017).

**Concretization** was perceived as being of great help in understanding the psychic and social realities participants were facing. The great majority recalled the activity in which the therapist concretized the cycle of violence. “The very moment I saw that circle enacted, the way violence kept repeating, I really understood what was happening to me” reports Diana (2012).

A few participants told us about the way the activities using **future projection** helped them experience their inner power: “I envisioned for myself the professional role I was going to have 5 years later. That was tough, but I managed to feel confident, capable and fulfilled,” says Mona (2012).

Intense experiences sometimes led to **catharsis.** If experienced, catharsis meant releasing and relief: “I felt liberated and so light” (Mona). Observing it as a witness led to fear and also to identification and a raw model of courage. As Maria describes: “…she had such a reaction... she got down on her belly and burst out sobbing. I got so shaken... I was also scared... how can I put it... that shook me deeply and eventually I was happy she could do that, and I couldn’t... because of so much that was shut up and restrained, I couldn’t manifest like that, although I was doing it in my heart” (Maria, 2017).

### Theme 3: The Process of Change

This theme offers an insider’s perspective of the process of change, as it was felt, experienced and explained by the study participants. It represents “the participants’ voice” which answers the question of *how* changes occurred in their lives, starting from the group experience.

#### **3.1.** The Basis of Change – The Perceived Effects of Psychodrama Intervention

Once they were “taken into care” by the therapist and as the group was organizing and becoming cohesive, and while they experienced trust, safety, respect and revaluation, women built a base for change.

Breaking of silence presupposes overcoming shame and the courage to talk. “And when I saw the others in the group I thought ‘Oh, God’ how many women I see suffering and I thought: away with the shame, it’s no use, it just makes me sink more, I went deeper from where I wanted to get out, and acceptance intervened in time” (Maria, 2012). For all participants, telling their story of a life filled with violence not only means beating the shame, but is also a great act of courage and power. Those who succeeded became raw models for the others: “Their power (the colleagues’) helped me open up too…” (Mona, 2017).

##### Releasing and relief

Breaking silence provides a feeling of releasing the pain, of relief. All participants had moments of releasing the negative emotions accumulated over time. “I remember that once I cried here, I also cried at home and all that crying made me feel good, all that pain... it takes out all the pain and I felt lighter the next day, stronger the third day, like when you get out of the morass and take a shower. Yes, that smell of mud doesn’t go away that easily and I felt lighter…” (Maria, 2012).

##### Understanding the pattern/insight

Though participants became aware of the pattern conveyed during their childhood, the level of understanding and acceptance differs. One participant says: “I’ve got the awareness that this issue of self-mistrust and lack of forgiveness for myself actually came from my childhood... I don’t know and I didn’t even spend time thinking whether that caused my marriage failure... because... that would hurt a lot (Erna, 2017). Another was able to go deeper: “...and I realized while I was coming here (to the group) that I have to clean up inside myself so that things fall in place around me”. She goes on to say: “I understood and also accepted… that problems come from my childhood, from a bad family model, from a model of wife and mother which was handed down to me as being very important, it was the best my mother could do, what can I say...” (Maria, 2012).

##### Hope and optimism

Sharing life stories enabled participants to identify and compare themselves with the other women. In most cases, this comparison led to the conclusion “I’m not the only one having problems” (Mioara, 2012) and “my problems are not even as big as the others”’ (Paula, 2012). Those findings, and also the activities designed to activate inner resources and re-build self-image, engendered feelings of trust, hope, power and determination to make changes and go further: “…They would fill me with optimism, I mean look at them with their tough problems and they continue to fight, and they have such drive, they don’t let themselves be dragged down...” (Erna, 2012).

Courage, as an inner resource activated by the group experience, figures in all participants’ remarks. “I had no idea of this courage inside myself […]When I came here I realized that was courage, I defined that myself, ultimately that is courage, no matter what...” (Maria, 2012). Courage and power occur together in all participants’ accounts.

##### Empowering and the determination to take action

For the great majority, one of the greatest benefits of participation in the group proved to be power. Initially, the power to hold on, as in Erna’s case (Erna, 2012): “… it was like a rescue, as if I was receiving power, no matter what the theme of the session.” It was the power to fight and finally solve things and change. More participants understood that “if you have a problem, don’t hide it under the carpet, because it will come after you eventually, as it is not solved” (Erna, 2012). The power they have achieved allowed many participants to face reality, no matter how painful that would be, to accept and assume pro-active behavior: “I realized I wasn’t the only one who has problems, that others have problems too, but only facing the situations will help you make some changes in your life” (Paula, 2017). In other cases, the benefit came from the newly found capacity, practiced within the group, to see the situation from another perspective, which gives space for hope and trust that there are solutions: “Yes, and I saw it differently (the situation), not necessarily better, but as I could detach myself from it, it wasn’t mine anymore and thus I could see the solution.” (Erna, 2012).

#### **3.2.** Relations

##### **3.2.1.** The relationship with one’s daughter or son

With one single exception, all participants who are mothers speak about changes in the relationship with their children. If the role of being a mother previously made them stay in the abusive relationship, a decision heavily influenced by the fear that they would not be able to raise the children on their own, this role later gave them power, strength and motivation to fight and change the way they related to their children. This was especially true after they became aware that the victim role had been handed down to them transgenerationally: “I realized that I gave my children a poor model of what it means to be a woman, wife, mother, as I took it from my mother or from generation to generation and I went into it in the wrong way, and I had to make a change… (Maria). Most of them initiated the change since the group intervention: “I started with my daughter… by beginning with small moments, with reverberations until you arrive at important life events, I applied these...” (Maria).

In most cases, not only did the participants stop transmitting the victim model, but they also started to build and convey another model started to be built and delivered, one based on understanding, communication and expressed love, self-confidence and respect. Maria says: “I saw that if I love myself and respect my moment, the child will also respect that, and I felt good, she felt good, I had my moment, it was perfect both for me and for her” (Maria, 2017). Personal change generated changes in the way they related to their own children: “I feel the change in that I don’t want to be like her (mother), and […] my principle is to be closer to the children, to show them love, as this is what I missed” (Paula, 2017). Improved communication with self and others is also reflected in the quality of the relationship with the children: “This is what the group mostly taught me… to stop hiding. […] and now I try to talk as much as I can with him and with myself” (Erna, 2017).

In one case, the relationship with the child stayed on the same coordinates after 5 years. In 2012, Mioara said: “I wished she (mother) could have understood this need to not live with an alcoholic father… but […] I am living the same issue in turn, I also have a girl, I am supposed to take care of her and her needs.” Five years later, she reports how she reproduces the model in which she was raised herself: “anyway I always had this aggressiveness, often uncontrolled, I beat her when it was hard for me…after that I felt sorry.”

##### **3.2.2.** The relationship with one’s mother

Most of the participants also made changes in the mother-daughter relationship. Most of the women in the group claim that the intervention helped them understand their mothers better. They understand their mothers were victims themselves: “…she also inherited it from grandmother (the role of victim) … that’s what she saw at home (violence).” In this way, they gave meaning to their mothers’ powerlessness to get out of the abusive relationship, and to protect them from experiencing the abuse. What helped most of them was: “To have the opportunity to speak to her (during the group activities), as I’ve never talked with my mother about certain aspects and pain, hers and mine...” (Mona). Another woman said: “I was able to put these issues (on the scene) so as not to bother me anymore” (Paula, 2012), and after 5 years she says she feels “this reconciliation… she has been trying to understand her and come to grips with what the situations were then” (Paula, 2017).

Maria’s account describes the process of restructuring the relationship with her mother: “I managed 1 year after the group ended, by repeated trials... we both cried, we also fought… but the result was that I managed to talk to her and really discuss things”. The process was difficult, but “I was left with that (from the group), if it was hard for me to play her role for several minutes, she is the one who lives it, I wonder how she feels, how are things for her? And that helped me to keep going.” Nowadays, “it is not a very close relationship, yet we are not as far from one another as we were...it is a decent relationship, marked by peace, calm, and respect and... it’s a great achievement for me...” (Maria, 2017).

In a single case, both women, mother and daughter, find themselves in the same vicious circle: “She still judges me for not being able to choose well. And I wonder who generated this entire situation? Isn’t that my mother? Somewhere, she was always wrong. Tolerance for alcohol; this is the mistake… and it is also mine. I took it over” (Mioara, 2017).

##### **3.2.3.** The relationship with one’s life partner

Participants progressed in different ways over the 5 years. To some, progress meant experiencing relations in which they faced the old patterns. Talking about the moment she made the decision and had the power to break out of the addictive relationship, Ina refers to the group experience: “I think that the moment people talk, as happened in the group, the brain registers it anyway, it keeps the information there and shows it when needed and then you react to this kind of thinking…” and she continues “… the moment he was aggressive to me, something broke inside” and “I could see my past, I could see my father or my grandfather in the past and what they did when they were drunk and I said NO” […] At present, her decision is to become independent and enter a relationship which is free from the past conditioning. “This is how I see things, it is good to go out with people you resonate with, yet don’t try to save them, I think I practically tried to save this guy (the lover), because I may have wanted to save my father, and I couldn’t…and there’s nothing there to save, if he doesn’t want to save himself, going there and trying is pointless…” (Ina, 2017).

Maria, after a period of time spent by herself, had an 8-month relationship. “I’ve seen what it means to be loved, to be given things, to receive and not only to give, to be respected, I saw the effects of saying no, of setting limits, I saw that if I set some limits, he respects that, he doesn’t mind and even if he does mind he makes an effort to understand… I saw what it means to be treated with dignity” (Maria, 2017).

During those 5 years, the other three participants made smaller changes in this role: Mona gave up a relation that did not fulfill her, Erna, although in a new relationship, feels that “he is a good man… yet I don’t know why I can’t be more involved,” and Paula, the only one who stayed with her husband, says after 5 years: “he’s better, I understand him better… I’m calmer… he still upsets me.” Yet Mioara, although she managed to leave her alcoholic and violent husband – “I told him it was over. I could make that difference” – entered another relationship, again with an abusive man who is also an alcohol addict: “I am aware of the fact that I slipped into the old pattern again and I ended up, maybe without realizing it, by taking back that door mat” (Mioara, 2017).

### Theme 4: The Corollary of Change

This theme describes *where* the participants arrived 5 years after the group work ended and what significance they assign to the group psychodrama intervention.

#### **4.1.** Decisions Made Over 5 Years

During the 5-year period, all the participants faced having to make important decisions. For most of them, the first and most important decision was to stop being a victim; it was a decision they had made 5 years ago, during the group intervention. As Maria (2017) asserts, “I won’t be a Cinderella again! Because a woman, I’ve learned, wasn’t born to be a Cinderella. She was born to be respectable, to be loved, to love, to stand up, to live with dignity, not covered in ashes.”

Some of the women decided to separate from the abusing partner, others decided to try a new relationship, yet others decided to re-evaluate their social relations and to enjoy having new people around. For two of the participants, the social network is being re-structured after 5 years. While Ina considers that changing her social network is a consequence of growing up: “…I think it is normal, in one’s thirties to have other interests and another circle of friends” (Ina, 2017), to Maria, the whole restructuring of the social network was a part of the long and difficult process of change: “After the experience I had (of victimhood) and all that I’ve learned in this group, I decided to leave everything behind me, to clean up my life, to get rid of toxic people, false friends and... to start a new life.”

They all report that their decisions in the professional sphere led to better jobs. For some participants, success in their career became one of their greatest achievements as well as one of their greatest resources (Maria, Mona, Ina). Mona claims: “I’ve learned to have courage to want more; because I am competent. I am now just where I wanted to be (a psychologist)” (Mona, 2017). For Mioara, the job is the same challenge as it was 5 years earlier: she can’t stay longer in one place, so she leaves.

Participants had to make important decisions concerning where they live. Some of them changed their living place: Maria made a radical decision and moved to the countryside, 80 km away from her old home, starting a new life, while Mona started building her own business.

#### **4.2.** Self-Perception

For two of the participants, the group experience meant the beginning of a transformation they perceive as fundamental. In 2012, Ina found herself in an addictive relationship with a man whom she also worked with, and she feared being on her own, while in 2017 she claims: “…I want to say that I have a completely different life right now […] I’m organizing things on my own, I’m not expecting a man’s help, I mean I can do it and I’m not afraid anymore to do it alone.” Her plans for the future are: “Well, I even have starting my own business in mind.” Maria defines herself by the progress she made: “…starting from here, from the group, I raised myself and the rest came… like with dominoes: dignity, then power, then I found out what it means to be respected by those around me, because when I respect myself and feel worthy of love, I really am loved and respected […] I am aware now that I am a valuable woman.” And she continues: “yes, I am a happy woman…it’s not about an ideal happiness, it’s about living your daily life beautifully” (Maria, 2017).

For three other women, those 5 years, despite certain difficulties, also meant development, growth. While before the group intervention, Paula described herself as being: “...kind of introverted, I used to torment myself inside… and I revolted,” afterward she says: “now I’ve learned to be myself first of all, so that they may also see me as I am […] more communicative and I can say what I feel…”. Mona also talks about trust, authenticity and the courage to make decisions and accept the results: “I started to grow even then (during the group period). Now I have trust and courage and power to do things.”

Erna (2012) described herself as: “generally pessimistic, disappointed in myself because of the failures I’ve had…”. Concerning the group experience, she says: “I think I’ve woken up…it finished too soon for me, I think I would have needed more time...I felt like I climbed up the stairs, even if I immediately add that I haven’t solved everything… yet I feel this growth...”. When referring to the last 5 years, Erna says that: “I could even boast by saying that when I was hopeless or I thought I couldn’t make it anymore or that it was too much and too difficult, I remembered those times (the group) when I had such drive and trust...” (Erna, 2017).

Only one participant reports that after 5 years things are worse than before. “I hurried up toward something else, I walked on the same pattern…and I couldn’t take it anymore... so I went lower... (Mioara, 2012).” She has remained the prisoner of old patterns.

#### **4.3.** The Significance Assigned to the Psychodrama Intervention

When asked to summarize the group psychodrama intervention experience in a few words, all participants, no matter what their journey during the last 5 years was like, talked about getting help and the feeling of not being alone anymore: “It has helped me a lot to understand that I was not the only one who has troubles (Mioara, 2017)”; they also mentioned trust, safety, power and hope.

The significance they found in the group psychodrama experience refers to the traces, deep or less so, left in every participant’s life and destiny. To Erna, “it was like a ray of light, I held on to something, I became more aware of certain issues.”

For Ina, it was “a part of evolution… of releasing my inner demons.” Maria speaks about the way in which, through the psychodrama experience: “I have built myself, I’ve got rich…, I’ve finally made that jump on the trampoline, I was able to evolve, to get up and see the world…, and it’s so beautiful, seen from above, because if one stays in the dust, they think that’s normal, being in the dust.” Most participants feel something that Erna (2017) best articulates: “I really thank God I’ve got that chance.”

## Discussion

Contrary to our expectations, the findings from the questionnaires administered at the end of the psychodrama intervention program show no significant difference between the experimental and the control group as regards spontaneity, and differences that reached only marginal statistical significance for wellbeing.

However, the findings show a positive trend of improvement for both samples, indicating that both psychodrama and ecological interventions are able to support abused women in their recovery process. In the psychodrama group, quantitative results show that participants experienced a decrease in symptoms and problems (Problems scale), a reduction in risk behaviors (Non-risk items), and an improvement in wellbeing (total score). Building on this basis, the qualitative findings are meant to offer an insider’s view into the perceived impact of the psychodrama intervention program focusing on empowerment.

This study is innovative in the following respects: first, it is a longitudinal long-term follow-up of an intervention designed to offer psycho-social support to victims of gender violence; second, it provides an insider’s view and in-depth analysis of the change process stimulated by an empowering-oriented psychodrama intervention program; third, it proposes a three-path process of change typology.

The findings shed light on *where* the women started their recovery (from the victim role), *what* was helpful (therapeutic alliance, action methods and techniques, specific psychodrama activities), *how* change occurred and *where* the participants are now in their lives (5 years from the psychodrama group intervention), as a corollary of change.

With only one exception, all participants have “families haunted by violence” (Muntean and Munteanu, 2011, p. 70) and are third-generation victims. The meaning they ascribe to taking over the victim role contains elements of the traditionalist perspective and religious motivations. From generation to generation, mothers raise their daughters to accept subordination and male oppression (McLeod, 2015), including aggression. On the other hand, progress in social awareness, social policy and social service development (Dima and Beldianu, 2015), although slow, creates a more helpful context for abused women to put an end to the generational model. Psychodrama helped them recognize and understand their victim role and, most importantly, their co-responsibility for their own subordination and destiny of violence (Testoni, 2008), and thus initiate changes. In that sense, psychodrama as group therapy can offer an alternative social framework for change.

For all participants, the idea of participating in a group was accompanied by anxiety, shame and lack of trust. All of them had difficulty receiving help (Van der Kolk, 2015), a common theme being the belief that “no one and nothing can help them.”

All women acknowledged the key role played by the therapeutic alliance in helping them to begin developing a sense of trust and safety. The need for a gentle touch (Kellermann and Hudgins, 2000) reported by other studies is metaphorically expressed by one participant: “(the therapist)...came close to us in steps of smoke” (Maria). Results show how the relationship with the therapist becomes a new model of relationship that is based on respect, being valued, unconditional acceptance, care and trust. The issues of building trust and safety came across as very challenging for all participants. Building a secure base (Bowlby, 1969) is crucial and must be the foundation for all interventions (Van der Kolk, 2008).

For all women, the psychodrama group experience offered a “culture of non-condemnation and non-blame” (Redondo et al., 2009, p. 6), which gave them the courage to share their life stories and thus break the silence. Abused women experienced the power of therapeutic group factors, especially of universality (the common factor – abuse), hope, catharsis and corrective recapitulation of the primary family group through psychodrama scenes. Group cohesiveness, altruism, socializing, and interpersonal learning were also important (Yalom and Leszcz, 2008).

The findings indicate that the psychodrama therapist fulfilled the function of containing double (Hudgins, 2007) and mirror for victimized women, thus creating the conditions for the fusion and individuation experiences. The ‘double’ technique made it possible to emphasize the feeling of belonging and sharing others’ inner contents (Dotti, 2002).

Some of the women appreciated the positive impact of the empowering mirror in regaining the feeling of personal value, just as psychodrama literature emphasizes the role of the mirror in activating the observing self (Kellermann, 2007), challenging the participants to self-observation, and facilitating awareness. During the psychodrama sessions, women have the occasion to mirror themselves in the group, leading to identification and recognition. The findings show that most women recognized and took on the role of victim. Mirroring also allowed them to recover some parts of the self which had been lost in experience by the victim – such as courage, dignity, and self-worth – and helped them in taking steps toward assuming new roles. This is consistent with Kellermann’s observation that our true self mirrored – “validating mirroring” – “allows the blossoming of the true self” (Kellermann, 2007, p. 91).

The psychodrama techniques which were appreciated by the participants as having the greatest impact were role reversal and role playing. Role playing, an expression of the holistic person, one of the mechanisms of change (Boria, 1997), was highly appreciated because of its ability to activate positive emotions. Van der Kolk (2015) found that role playing can reactivate brain areas dedicated to pleasure, which have been rewired under stress and trauma.

Role play leads to the development of autonomy (Dotti, 2002): from being dependent persons, “stuck in a role,” they learn to take on new things – initially in semi-reality, in plus reality, in the secure environment of the group and then in the reality of everyday life (e.g., Maria confronted her mother; Erna her ex-husband; Paula her psychologically abusive partner). The findings are in agreement with Dayton’s argument (Dayton, 2013) that, to reduce the anxiety and newness of a role, it should be practiced in a safe environment. Role play may have promoted improved capacity for cognitive processing and action within relations, as shown by Versari (2014).

Role reversal was considered by all the abused women to be the most difficult experience. In some cases, it generated an intense emotional catharsis. Women in the psychodrama group experienced both facets of this catharsis, its healing power and potential for re-traumatization, which is acknowledged in the literature on abuse (Kellermann and Hudgins, 2000; Hudgins and Toscani, 2013). This risk was addressed by strictly respecting the structure of a psychodrama group session: warm-up, psychodramatic group or protagonist work and sharing. For example, all participants felt that psycho-motor activation at the beginning of the sessions and sharing emotions and rituals at the end were very useful. Integrating attachment theory into psychodrama group work emphasized the attention paid by the psychodramatist to the four core needs and related processes: safety (therapist, guarantor of safety), comfort (e.g., warm-up activities to reduce anxiety, sharing), regulating proximity (role playing, family social atom), and predictability (session structure, ritual activities) (Baim, 2014).

Role reversal was what helped participants gain insights and activated their spontaneity, flexibility and creativity, leading eventually to a perceptive decentering and the possibility of creating new roles. Most of the women gained a significantly better understanding of the other, and a better understanding of themselves. According to Kipper and Ritchie’s (2003) meta-analysis, role reversal and double are the most effective techniques of psychodrama.

The encounter technique allowed the participants to experience the tele in the psychodrama group to train their empathy (Kellermann and Hudgins, 2000; Dotti, 2002) and to discover authenticity in encountering their peers. For some of them, authentic relationships start to take the place of transferential relationships. As the self is made up of the roles we play (Moreno and Moreno, 1975), the findings show that some women restructured their roles and identity. Five years after the completion of the psychodrama program, a significant proportion of the women manage to self-describe as authentic people and attribute this fact to the experience of the psychodrama group (Paula, Maria, Mona, Ina).

To conclude, we could say that psychodrama, as an action method, has the potential to stimulate action in women’s lives and initiate adaptive coping strategies leading to resilience. This is consistent with Sung-Hee’s (2009) findings that psychodrama has a significant effect on helping domestic violence victims get ready to make practical changes.

Our findings show that the participants’ path after the end of the psychodrama group intervention was neither linear nor quiet. Moreno explains that conserving an old role, such as the victim role, might have inhibited change, but simultaneously provided a sense of stability and security. A warming up phase thus stimulates a spontaneity state (status nascendi), necessary for the development of new, creative solutions (creative phase) (Moreno, 1980). Schacht (2007, p. 37) calls attention to the fact that people “will probably have to cycle through the phases of the spontaneous-creative process a couple of times unless their new roles are stable enough”.

On the other hand, Pourtois et al. (2013, p. 60) develop a scheme for interpreting the resilient phenomenon. They propose a model that integrates the analysis of post-traumatic human behavior with concepts of resilience, resistance, non-resistance and non-resilience. The four concepts constitute “escape strategies used by people who have suffered a major trauma.”

Thus, our in-depth analysis of women’s starting points – the victim role, change processes and outcomes over 5 years – indicated that change takes place along three paths, which differ according to the type of change in the victim role and the victim’s resilience. These paths are the basis for our proposed typology:

(1) **Proactive – Resilient type** (Maria, Ina): shows high resilience, feels inner strength and self-esteem (re)gained, acts based on her own initiative, makes decisions, proposes and makes changes in her own life, has sufficient determination to create favorable contexts and to act toward achieving own goals; over time, she rebuilds her role as a partner, mother and daughter. Self-perception changes significantly, it is reinvented; the reconstruction of identity can be the crowning achievement of this change. Is aware of the victim model and transmission pattern and has the power to put an end to the *trans*-generational transmission.
(2) **Active – Resistant type** (Paula, Erna, Mona): although this type shows resilience, the strength in fighting the problems of life prevails; the woman comes into contact with her inner strength, improves her self-image, is active, shows improvement in some areas of psychological and social functioning; enacts some changes in her personal life; can enact changes in some roles – as mother and daughter, for instance – and makes adjustments in her role as partner. Has a vacillating evolution with possible stagnation or even relapse. Has some awareness of the victim model and its transmission pattern, may put an end to the trans-generational transmission.
(3) **Repetitive – Non-resilient type** (fr. *desilience*) (Mioara): no substantial changes in the role of partner, mother, daughter; continues to perceive human relationships in the form of submission and dependency; on a personal level, has feelings of desperation and alienation; has a high risk of seeking refuge in religious cults/groups. Has some awareness of the victim model and transmission pattern but cannot put an end to it and preserves her main role as a victim.

If we agree that resilience is a dynamic process (Ionescu, 2013), we agree that each woman can navigate from one category to another, at different points in time, depending on the complex interplay of internal and external factors.

### Limitations of the Study

The typology presented here is based on data from 6 abused women and is valid for this sample. It offers a framework for understanding the change path and can be useful in calibrating intervention services to reflect the characteristics of each type. However, we can make claims to generalizability, and further research is needed to verify this typology.

Another limit is the small convenience sample, where participants were assigned to the experimental and control groups on a voluntary basis, rather than randomly. Thus, it is possible that participants volunteering for the experimental group were more resilient and open to change, or in another phase of their healing process. Also, the possibility that several of the reported effects occurred by chance cannot be ruled out.

Follow-up was possible for only one of the two psychodrama groups, consisting of women living at home. Exploring the experiences of sheltered women could emphasize different aspects. In addition, women participating in the control group could not be tracked and there are no follow-up data for this group.

To conclude, this study does not aim to be representative, as its main objective is to explore processes in-depth and find meanings.

## Conclusion

The study sought to demonstrate the value of psychodrama in working with abused women and show how methods and techniques can empower them and stimulate changes in their victim role.

In working with women victims of abuse, the therapeutic relationship is fundamental to the intervention, and must be built slowly and gently to develop a sense of trust and safety. In addition, the psychodramatist must be aware and careful when assuming the roles of double and mirror for the abused women. The psychodrama psychotherapist has an active and proactive role, avoids a neutral approach and favors a direct, authentic human relationship experience that can be configured as positive. Our findings indicate that the good therapeutic relationship entailed a balanced activation of the three functions named by Moreno (1953) – producer, therapist, and analyst.

The most helpful techniques in changing the victim role were role reversal, role playing, empowering mirror and concretization. The women who showed the greatest change after 5 years considered that role reversal was the most impactful experience and helped them initiate the changes they made. On the other hand, all participants perceived role reversal as being the most difficult experience, which draws attention to the risk of re-traumatization. We recommend that it be used carefully, mindful of both its benefits and risks: its ability to activate the spontaneity and creativity needed to create new roles, and the danger that it can re-traumatize.

We could say that psychodrama, as an action method, has the potential to stimulate action in women’s lives and initiate adaptive coping strategies leading to resilience. We identified three paths or directions of change for women who participated in an empowering-oriented psychodrama intervention program. On the basis of these paths, we propose a typology: the Proactive – Resilient type shows high resilience, significant changes in roles and is capable of ending the trans-generational transmission of the victim role; the Active – Resistant type can enact changes in some roles and may put an end to the *trans*-generational transmission; the Repetitive – Non-resilient type, with no substantial changes in roles, remains caught in the victim pattern. As women can navigate from one category to another, services should be calibrated accordingly.

Our findings allow us to suggest that specialists and providers of assisted resilience to victims of gender violence can build trust and safety, a nurturing and caring attitude, and activate intra-psychic strengths and empowerment by using role-play in a secure environment to practice new, feared or wished roles. Therapists working with abused women, either in groups or as individuals, can use psychodrama techniques such as empowering mirror, validating mirror, containing double, concretization, role-play and role-reversal.

To minimize the risk of re-traumatization, professionals should consider an empowering-oriented psychodrama model in the initial phases of working with abused women, until they are strong enough and prepared for psychodrama therapy. In addition, it is essential that therapists respond to women’s need for safety and comfort through warm-up activities (which reduce anxiety) and sharing, as well as the need for predictability through the session structure and ritual activities.

Among its strengths, this study provides an insider’s view into the process of change experienced by women participating in a psychodrama intervention program and proposes a three-path typology of the change process. While no claims to generalizability can be made, future research could verify this typology.

Including psychodrama among the psycho-social services offered to abused women can be a valuable contribution, especially as regards empowerment, role-reconstruction and interruption of the *trans*-generational pattern.

## Ethics Statement

The 2011–2012 Empower Daphne Project was carried out in accordance with guidelines of the University of Padova Scientific and Ethics Committee. The protocol was approved by the University of Padova Department of Applied Psychology Scientific and Ethics Committee in 2010. All subjects gave their written consent in accordance with the Declaration of Helsinki.

## Author Contributions

All authors listed have made a substantial, direct and intellectual contribution to the work, and approved it for publication.

## Conflict of Interest Statement

The authors declare that the research was conducted in the absence of any commercial or financial relationships that could be construed as a potential conflict of interest.

## References

[B1] Adorjani J. (2012). *The Perception Of Women Victims Of Domestic Violence Regarding The Crim^∗^Inal Justice System.* Ph.D. thesis, Babes-Bolyai University Cluj-Napoca.

[B2] Baim C. (2014). “Integrating psychodrama with attachment theory. Implications for practice,” in *Empowering Therapeutic Practice: Integrating Psychodrama into other Therapies* eds Holmes P. Farrall M. Kirk K. (London: Jessica Kingsley Publishers) 125–156.

[B3] Boria G. (1997). *Lo Psicodramma Classico.* Milano: Franco Angeli.

[B4] Bowlby J. (1969). *Attachment and Loss: Volume 1. Attachment.* New York, NY: Basic Books.

[B5] Brocki J. M. Wearden A. J. (2006). A critical evaluation of the use of interpretative phenomenological analysis (IPA) in health psychology. *Psychol. Health* 21 87–108. 10.3109/09638288.2014.939770 25009949

[B6] Bucuţă M. Dima G. Zoltani K. Antal D. D. (2012). The phenomenon of domestic violence in Romania: a prevention and intervention. *Int. J. Fam. Stud.* 17 153–168.

[B7] Coleman E. J. Sandfort T. (2014). *Sexuality and Gender in Postcommunist Eastern Europe and Russia.* New York, NY: Routledge.

[B8] Council of Europe (2011). *Convention on Preventing and Combating Violence Against Women and Domestic Violence, No. 210.* Available at: http://www.conventions.coe.int/Treaty/

[B9] Creswell J. W. Plano Clark V. L. (2018). *Designing and conducting mixed methods research* 3rd Edn. Los Angeles, CA: Sage.

[B10] Dayton T. (2013). *Psychodrama and the Treatment of Addiction.* Available at: https://www.tiandayton.com/wp-content/uploads/2013/03/Psychodrama-and-the-Treatment-of-Addiction-and-Trauma-in-Women.pdf

[B11] Dima G. Beldianu I. (eds) (2015). *Violenta Domesticã: Interventia Coordonatã a Echipei Multidisciplinare: Manual Pentru Specialişti.* Timişoara: Editura de Vest.

[B12] DiQuinzio P. (1999). Exclusion and essentialism in feminist theory: the problem of mothering. *Hypatia* 8 1–20. 10.1111/j.1527-2001.1993.tb00033.x

[B13] DiQuinzio P. (2013). *The Impossibility Of Motherhood: Feminism, Individualism And The Problem Of Mothering* 2nd Edn. New York, NY: Routledge.

[B14] Dotti L. (2002). *Lo Psicodramma Dei Bambini. i Metodi D’azione in Età Evolutiva* Terza Edn. Milano: Franco Angeli.

[B15] Elliott R. Fischer C. T. Rennie D. L. (1999). Evolving guidelines for publication of qualitative research studies in psychology and related fields. *Br. J. Clin. Psychol.* 38 215–229. 10.1348/014466599162782 10532145

[B16] European Union Agency for Fundamental Rights [FRA] (2014). *Violence Against Women: An EU-Wide Survey. Main Results. Publications Office of the European Union. Luxembourg. 2014.* Available at: http://fra.europa.eu/sites/default/files/fra-2014-vaw-survey-main-results-apr14_en.pdf

[B17] European Women’s Lobby (2017). *Violence Against Women and Girls: Will Europe Rise Up in 2017?* Available at: https://www.womenlobby.org/will-Europe-rise-up-to-end-VAWG?lang=en

[B18] Evans C. Connell J. Barkham M. Margison F. McGrath G. Mellor-Clark J. (2002). Towards a standardised brief outcome measure: psychometric properties and utility of the CORE-OM. *Br. Journal of Psychiatry* 180 51–60. 10.1192/bjp.180.1.51 11772852

[B19] Faludi S. (1991). *Backlash: The Undeclared War Against American Women.* New York, NY: Doubleday.

[B20] Hudgins K. Toscani F. (eds) (2013). *Healing World Trauma with the Therapeutic Spiral Model.* London: Jessica Kingsley Publishers.

[B21] Hudgins M. K. (2007). “Clinical foundations of the therapeutic spiral model: theoretical orientations and principles of change,” in *Psychodrama: Advances in Theory and Practice* eds Baim C. Burmeister J. Maciel M. (East Sussex: Routledge) 175–188.

[B22] Ionescu S. (2013). *Tratat de Rezilienţã Asistatã.* Bucureşti: Editura Trei.

[B23] Kaplan C. (2001). Hillary rodham clinton’s orient: cosmopolitan travel and global feminist subjects, meridians: feminism, race. *Transnationalism* 2 219–240.

[B24] Kellermann F. (2007). “Let’s face it: mirroring in psychodrama,” in *Psychodrama: Advances in Theory and Practice* eds Baim C. Burmeister J. Maciel M. (East Sussex: Routledge) 83–96.

[B25] Kellermann P. F. Hudgins M. K. (eds) (2000). *Psychodrama With Trauma Survivors: Acting Out Your Pain.* London: Jessica Kingsley Publishers.

[B26] Kipper D. (1998). Psychodrama and trauma: implications for future interventions of psychodramatic role-playing modalities. *Int. J. of Act. Methods* 51 113–121.

[B27] Kipper D. A. Ritchie T. D. (2003). The effectiveness of psychodramatic techniques: a meta-analysis. *Group Dyn.* 7 13–25. 10.1037/1089-2699.7.1.13

[B28] Kipper D. A. Shemer H. (2006). The revised spontaneity assessment inventory (SAI-R): spontaneity, well-being and stress. *J. Group Psychother. Psychodrama Sociom.* 59 127–136. 10.3200/JGPP.59.3.127-136

[B29] Kruckenberg L. J. (2010). “Politics of difference and activism at intersections: romani women in eastern and central Europe,” in *The Politics of Gender. A Survey* ed. Lee Y.-L. (London: Routledge) 25–45.

[B30] Larkin M. Watts S. Clifton E. (2006). Giving voice and making sense in interpretative phenomenological analysis. *Q. Res. Psychol.* 3 102–120. 10.1191/1478088706qp062oa

[B31] McLeod J. (2015). “Gender identity, intergenerational dynamics, and educational aspirations: young women’s hopes for the future,” in *Handbook of Children and Youth Studies* eds Wyn J. Cahill H. (Berlin: Springer) 315–327. 10.1007/978-981-4451-15-4-6

[B32] Metcalfe B. D. Afanassieva M. (2005). Gender, work, and equal opportunities in central and eastern Europe. *Women Manag. Rev.* 20 397–411. 10.1108/09649420510616791

[B33] Meyers D. T. (2001). The rush to motherhood: pronatalist discourse and women’s autonomy. *Signs* 26 735–773. 10.1086/495627

[B34] Moreno J. L. (1953). *Who Shall Survive? Foundations of Sociometry, Group Psychotherapy and Sociodrama.* New York, NY: Beacon House.

[B35] Moreno J. L. (1980). *Psychodrama* Vol. 1 New York, NY: Beacon House

[B36] Moreno J. L. (2009). *Scrieri Fundamentale: Despre Psihodrama, Metoda de Grup şi Spontaneitate.* Bucureşti: Editura Trei.

[B37] Moreno J. L. Moreno Z. T. (1975). *Psychodrama, Volume One: Foundations of Psychotherapy.* New York, NY: Beacon House.

[B38] Muntean A. Munteanu A. (2011). *Violenţã, Traumã, Rezilienţã (Violence, Trauma and Resilience).* Iasi: Polirom.

[B39] Occhipinti L. (1996). Two steps back? Anti-feminism in Eastern Europe. *Anthropol. Today* 12 13–18. 10.2307/2783403

[B40] Pourtois J.-P. Humbeeck B. Desmet H. (2013). “Rezistenţã şi rezilienţã asistate: o contribuţie la susţinerea educativã şi psihosocialã,” in *Tratat de Rezilienţã Asistatã* ed. Ionescu S. (Bucureşti: Editura Trei) 58–79.

[B41] Redondo J. Antónia M. Cravador A. (2009). Victims of domestic violence, traumatic experience and morenian psychodrama. *Paper Presented at 17th I.A.G.P* Rome: Congress.

[B42] Reid K. Flowers P. Larkin M. (2005). Exploring lived experience. *Psychologist* 18 20–23.

[B43] Reteaua Pentru Prevenirea Si Combaterea Violentei Impotriva Femeii (2018). INFOGRAFICE: Violenţa în Familie în 2017 Conform Datelor Oficiale Ale Poliţiei. (INFOGRAPHICS: Domestic Violence in 2017 According to Official Police Data). Available at: https://violentaimpotrivafemeilor.ro/violenta-in-familie-in-2017-conform-datelor-oficiale-ale-politiei/

[B44] Saxonberg S. Sirovatka T. (2008). Seeking the balance between work and family after communism. *Marriage Fam. Rev.* 39 287–313. 10.1300/J002v39n03-04

[B45] Schacht M. (2007). “Spontaneity - creativity: the psychodramatic concept of change,” in *Psychodrama: Advances in Theory and Practice* eds Baim C. Burmeister J. Maciel M. (East Sussex: Routledge) 21–40.

[B46] Shaw R. (2001). Why use interpretative phenomenological analysis in health psychology? *Health Psychol. Update* 10 48–52. 10.1111/j.2044-8341.2010.02001.x 22903872

[B47] Smith J. A. (1996). Beyond the divide between cognition and discourse: using interpretative phenomenological analysis in health psychology. *Psychol. Health* 11 261–271. 10.1080/08870449608400256

[B48] Smith J. A. Flowers P. Larkin M. (2009). *Interpretative Phenomenological Analysis: Theory, Method and Research.* London: Sage Publications.

[B49] Sung-Hee C. (2009). Psychodrama and motivation for change for domestic violence victims. *Korean J. Psychodrama* 12 1–10. 10.17962/kjp.2009.12.2.001

[B50] Testoni I. (2008). *La Frattura Originaria.* Liguori: Napoli.

[B51] Testoni I. Armenti A. Bertoldo A. Di Lucia Sposito D. Wieser M. Moita G. (2013a). “Assessing psychodramatic intervention on female victims of violence. The cross-cultural validation of CORE-OM and SAI-R for project Empower Daphne,” in *Daphne and the Centaurus – Overcoming Gender Based Violence* eds Arcidiacono C. Testoni I. Groterath A. (Berlin: Barbara Budrich Publishers) 161–176.

[B52] Testoni I. Armenti A. Wieser M. Bertoldo A. Bucuta M. Tarashoeva G. (2013b). “The effectiveness of the EMPoWER project and intervention: psychodrama and the elaboration of domestic violence in Italy, Austria, Bulgaria, Portugal, Romania, and Albania,” in *Teaching Against Violence. Reassessing the Toolbox. Teaching with Gender. European Women’s Studies in International and Interdisciplinary Classrooms* eds Testoni I. Groterath A. Guglielmin M. S. Wieser M. (Utrecht: Atgender) 119–150.

[B53] The World Health Organization [WHO] (2002). *W^∗^orld Report on Violence and Health.* Available at: http://www.who.int/violence_injury_prevention/violence/world_report/en/summary_en.pdf accessed on 20/09/2015

[B54] Van der Kolk B. (2008). The compulsion to repeat the trauma. Re-enactment, revictimization, and masochism. *Psychiatr. Clin. N. Am.* 12 389–411. 10.1016/S0193-953X(18)30439-8 2664732

[B55] Van der Kolk B. (2015). *The Body Keeps the Score: Brain, Mind, and Body in the Healing of Trauma.* London: Penguin Books.

[B56] Versari A. (2014). Analytic psychodrama for overcoming interpersonal violence: a mixed group of victims and perpetrators. *EJ. Psychother. Res.* 4 Available at: http://psychotherapyjournal.org/analytic-psychodrama-for-overcoming-inter personal-violence-a-mixed-group-of-victims-and-perpetrators/?print=pdf

[B57] World Economic Forum (2012). *The Global Gender Gap Index 2012.* Available at: http://www3.weforum.org/docs/GGGR12/

[B58] Yalom I. D. Leszcz M. (2008). *Tratat de Psihoterapie de Grup. Teorie şi Practicã.* Bucureşti: Editura Trei.

